# Portal vein stent placement after hepatobiliary and pancreatic surgery

**DOI:** 10.1007/s00423-020-01917-9

**Published:** 2020-07-03

**Authors:** Ammar Khan, Dyre Kleive, Einar Martin Aandahl, Bjarte Fosby, Pål-Dag Line, Eric Dorenberg, Steinar Guvåg, Knut Jørgen Labori

**Affiliations:** 1 Department of Transplantation Medicine, Oslo University Hospital, Sognsvannsveien 20, 0372 Oslo, Norway; 2 Institute of Clinical Medicine, University of Oslo, Oslo, Norway; 3 Department of Hepato-Pancreato-Biliary Surgery, Oslo University Hospital, Oslo, Norway; 4 Department of Radiology, Oslo University Hospital, Oslo, Norway

**Keywords:** Portal vein, Interventional radiology, Pancreatoduodenectomy, Liver transplantation, Patency

## Abstract

**Purpose:**

To evaluate the long-term outcomes of percutaneous transhepatic stent placement for portal vein (PV) stenosis after liver transplantation (LT) and hepato-pancreato-biliary (HPB) surgery.

**Methods:**

Retrospective study of 455 patients who underwent LT and 522 patients who underwent resection of the pancreatic head between June 2011 and February 2016. Technical success, clinical success, patency, and complications were evaluated for both groups.

**Results:**

A total of 23 patients were confirmed to have postoperative PV stenosis and were treated with percutaneous transhepatic PV stent placement. The technical success rate was 100%, the clinical success rate was 80%, and the long-term stent patency was 91.3% for the entire study population. Two procedure-related hemorrhages and two early stent thromboses occurred in the HPB group while no complications occurred in the LT group. A literature review of selected studies reporting PV stent placement for the treatment of PV stenosis after HPB surgery and LT showed a technical success rate of 78–100%, a clinical success rate of 72–100%, and a long-term patency of 57–100%, whereas the procedure-related complication rate varied from 0–33.3%.

**Conclusions:**

Percutaneous transhepatic PV stent is a safe and effective treatment for postoperative PV stenosis/occlusion in patients undergoing LT regardless of symptoms. Due to increased risk of complications, the indication for percutaneous PV stent placement after HPB surgery should be limited to patients with clinical symptoms after an individual assessment.

## Introduction

Portal vein (PV) stenosis is a well-known complication after liver transplantation (LT), and hepato-pancreato-biliary (HPB) surgery [1–8]. Potential symptoms related to PV stenosis are abdominal pain, liver failure, intractable ascites, and gastrointestinal bleeding [9–11] which render it as a potentially serious condition. Management of postoperative PV stenosis with percutaneous stent placement has previously been reported to be a viable and less invasive option than laparotomy and surgical revision [12]. Even though PV stenosis is a rare complication after LT [2, 3], it has recently been shown to be as high as 19.6% after pancreatoduodenectomy [13]. Regarding LT, PV stenosis is more frequent after pediatric transplantation due to size mismatch [14].

With an increasing rate of surgical resection and vascular reconstruction in HPB surgery, an increasing incidence of complications related to the PV must be anticipated [15–17]. Percutaneous transhepatic stenting of the PV has gained acceptance as a safe minimally invasive procedure with promising results [12, 18–24]. The aim of this paper is to evaluate the results of stenting of PV stenosis after LT and HPB surgery within a high-volume tertiary referral center.

## Materials and methods

This was a retrospective review of all patients undergoing PV stent placement due to postoperative PV stenosis at Rikshospitalet, Oslo University Hospital between June 2011 and February 2016. The hospital is a tertiary referral center for all HPB surgery in the southeast health region (3 million inhabitants) and the only national center for abdominal transplantation surgery in Norway (5.3 million inhabitants). Hospital records, including radiological reports, were reviewed. Type of surgery, time from surgery to PV stent placement, and complications to the procedure were assessed. Patency was evaluated according to latest available radiological modality, as follow-up was heterogeneous. Methods for pancreatic resections and LT were performed as described elsewhere [25, 26]. The hospital review board approved the study (2016/8365) according to the general guidelines provided by the regional ethics committee. The manuscript was completed in accordance with the STROBE statement [27].

### Indication for stent placement

For patients undergoing LT, ultrasonography (US) on postoperative day (POD) 1 was performed routinely. Subsequent radiology was obtained on clinical suspicion of adverse events. Patients undergoing pancreatoduodenectomy were not subjected for routine radiology in the immediate postoperative period, but patients with concomitant PV resection had US on POD 1 and the day before discharge. The criteria for definite diagnosis of significant PV stenosis were a 3-fold increase in portal blood velocity detected by US and a minimum of 50% PV diameter reduction on contrast-enhanced computed tomography (CT) or percutaneous transhepatic portography. PV occlusion was defined as the absence of contrast enhancement through the PV. Patients with radiological significant PV stenosis underwent PV stenting either because of clinical manifestation or as a preemptive treatment in order to avoid future complications. Hence, indication for PV stent placement was based on radiological findings, clinical manifestations, or suspicion of future adverse events related to untreated PV stenosis.

### Stent placement procedure

Routinely, the procedures were performed with the patients under conscious sedation. In all patients, access to the PV was gained by percutaneous US-guided puncture of a subsegmental or segmental portal branch with introduction of a 6 Fr introducer (Merit Medical Systems, South Jordan, UT, USA). After diagnostic angiography with confirmation of significant stenosis, stenting was performed using self-expanding nitinol stents 40–60 mm in length and oversized in diameter by 1–2 mm as compared with the adjacent PV. For diameters of 10 and 12 mm, we used Smart Control self-expanding stents (Cordis, a cardinal health company, Dublin, OH, USA); in larger diameters, 14–20 mm Sinus-XL Flex stents (Optimed Medizinische Instrumente GmbH, Ettingen, Germany) were placed. In cases of residual stenosis, stents were dilated with balloons at the size of the prestenotic portal segment. During removal of the introducer sheaths, gelfoam pledgets were placed in the access channel in order to prevent hemorrhage. Technical success was defined as residual stenosis < 30% or < 10% difference in pressure gradient on immediate angiography and absence of significant stenosis on early follow-up at US or CT. After the procedure, the patients received prophylactic low molecular weight heparin based on their body weight (150 IU/kg/day) for a minimum of 3 months. Following PV stent placement, supplementary radiology in addition to routine follow-up was obtained on clinical indication.

## Results

Between June 2011 and February 2016, 455 patients underwent LT and 522 patients underwent resection of the pancreatic head (pancreatoduodenectomy *n* = 494, total pancreatectomy *n* = 28). There were 14 patients (3.1%) in the LT group and eight patients (1.5%) in the HPB group confirmed to have postoperative PV stenosis. Six of the eight HPB patients underwent resection and reconstruction of the PV during the primary operation. In addition, one patient developed postoperative PV stenosis after a resection of the *extrahepatic bile ducts*. An overview of the patients is presented in Table 1. All of the 23 patients with PV stenosis/occlusion underwent a technical successful percutaneous transhepatic PV stent placement. The etiology of PV stenosis was anastomotic stenosis in 17 patients while the remaining six were caused by tumor recurrence and/or thrombosis. Only 10 of 23 patients had clinical manifestations while the remaining 13 patients had an asymptomatic PV stenosis discovered on routine radiological follow-up. Eight of 10 patients with clinical manifestations experienced disappearance of symptoms after percutaneous transhepatic PV stent placement resulting in a clinical success rate of 80%. There were two procedure-related hemorrhages and two early stent thromboses in the HPB group resulting in a complication rate of 17.4% for the entire study population. Long-term stent patency for the entire study population based on the last available radiological imaging was 91.3% with a median follow-up of 6 months in the HPB group and 49 months in the LT group (Table 2).

**Table 1:** Overview of 23 patients receiving portal vein stent

No.	Histology	Surgery	Indication for PV stent	Resolvement of symptoms	Anticoagulation	Complications	Follow-up: stent to US/CT
1	Neuroendocrine tumor	Pancreatoduodenectomy	Ascites	Yes	LMWH 7500 × 1	None	926 days
2	Ampullary adenocarcinoma	Pancreatoduodenectomy	Ascites	No	LMWH 7500 × 2	Bleeding	33 days
3	Distal cholangiocarcinoma	Pancreatoduodenectomy	Thrombosis	No symptoms	LMWH 7500 × 2	Early stent thrombosis	282 days
4	Pancreatic ductal adenocarcinoma	Pancreatoduodenectomy	GI bleeding and ascites	Yes	LMWH 5000 × 2	Bleeding	176 days
5	Pancreatic ductal adenocarcinoma	Pancreatoduodenectomy	Ascites	Yes	LMWH 7500 × 1	None	1912 days
6	Pancreatic ductal adenocarcinoma	Pancreatoduodenectomy	Ascites	Yes	LMWH 5000 × 1 + lifelong aspirin	None	152 days
7	Pancreatic ductal adenocarcinoma	Pancreatoduodenectomy	Stenosis	No symptoms	LMWH 5000 × 2	None	510 days
8	Pancreatic ductal adenocarcinoma	Total pancreatectomy	Stenosis	No symptoms	LMWH 5000 × 2	None	114 days
9	Distal cholangiocarcinoma	Bile duct resection	Ascites	No	LMWH 5000 × 2	Early stent thrombosis	9 days
10	Non-malignant	Liver transplantation	Ascites	Yes	LMWH 5000 × 2 + lifelong aspirin	None	1462 days
11	Non-malignant	Liver transplantation	GI bleeding and ascites	Yes	LMWH 5000 × 1 + lifelong aspirin	None	1455 days
12	Non-malignant	Liver transplantation	Thrombosis	No symptoms	LMWH 5000 × 2	None	1184 days
13	Non-malignant	Liver transplantation	Stenosis	No symptoms	LMWH 5000 × 1 + lifelong aspirin	None	2262 days
14	Non-malignant	Liver transplantation	Stenosis	No symptoms	LMWH 5000 × 1 + lifelong aspirin	None	1813 days
15	Non-malignant	Liver transplantation	Stenosis	No symptoms	LMWH 5000 × 2	None	1963 days
16	Non-malignant	Liver transplantation	Stenosis	No symptoms	LMWH 5000 × 1 + lifelong aspirin	None	731 days
17	Non-malignant	Liver transplantation	Stenosis	No symptoms	LMWH 5000 × 1 + lifelong aspirin	None	2507 days
18	Non-malignant	Liver transplantation	Stenosis	No symptoms	LMWH 7500 × 1 + lifelong aspirin	None	1745 days
19	Neuroendocrine tumor	Liver transplantation	Stenosis	No symptoms	LMWH 5000 × 2 + lifelong aspirin	None	1797 days
20	Cholangiocarcinoma	Liver transplantation	Stenosis	No symptoms	LMWH 5000 × 2	None	554 days
21	Cholangiocarcinoma	Liver transplantation	Stenosis	No symptoms	LMWH 5000 × 1 + lifelong aspirin	None	25 days
22	Cholangiocarcinoma	Liver transplantation	Ascites	Yes	LMWH 5000 × 1 + lifelong aspirin	None	No US/CT
23	Cholangiocarcinoma	Liver transplantation	Ascites	Yes	LMWH 5000 × 1 + lifelong aspirin	None	187 days

**Table 2:** Baseline characteristics and summary of periprocedural outcomes

Baseline characteristics	All	HPB surgery	Liver transplantation
Number	23	9	14
Age, median (range)	58 (33–74)	62 (47–74)	47 (33–68)
Sex (male/female)	15/8	6/3	9/5
BMI, median (range)	23 (18–31)	24 (18–28)	22 (19–31)
Child-Pugh classification (A/B/C)	15/4/4	9/0/0	6/4/4
Etiology for PV stenosis			
Tumor recurrence	4	2	2
Anastomotic stenosis	17	5	12
Postoperative thrombosis	2	2	0
Indication for PV stent			
Ascites	8	5	3
GI bleeding and ascites	2	1	1
Asymptomatic	13	3	10
Resolvement of symptoms			
Ascites resolved	6	3	3
GI bleeding and ascites resolved	2	1	1
Symptoms not resolved	2	2	0
Time from primary operation to stent, median (min-max), days	177 (21–1565)	237 (43–1565)	163 (21–937)
Time from stent placement to last CT, median (min-max), days	828 (9–2507)	176 (9–1912)	1462 (25–2507)
Complications of PV stent	2/23	2/9	0/14
Early stent occlusion < 30 days	2/23	2/9	0/14
Patent stent on last CT	21/23	7/9	14/14

## Discussion

In this study, we investigated the outcome of PV stent placement for postoperative PV stenosis after LT and HPB surgery. PV stent placement was performed with a procedure-related complication rate of 17.4% for the entire study population, confirming the results of previous publications [11]. The technical success rate was 100%, the clinical success rate was 80% among patients with clinical manifestations, and the long-term stent patency was 91.3% for the entire study population. This shows that PV stenting is effective in relieving PV stenosis after LT and HPB surgery. The results of this audit are comparable with other publications; [12, 24, 28] however, comparison must be done with caution as there are obvious inter-institutional differences in the postoperative follow-up and indication of PV stent placement. In our institution, patients with clinical and/or radiological signs of significant PV stenosis were subjected to PV stenting after evaluation by a multidisciplinary team. To the best of our knowledge, there are few studies investigating which patients benefit from postoperative PV stent placement.

Among the patients with PV stent placement after LT, there were no procedure-related complications and a long-term patency of 100%. This supports the decision to preemptive treat asymptomatic patients with radiological signs of significant PV stenosis after LT to reduce the risk of future complications. Importantly, PV complications after LT have been well documented because PV stenosis and thrombosis can potentially be devastating and lead to graft failure [14]. PV stenosis is more frequent after pediatric LT, and some centers have practiced an early approach to PV abnormalities during pediatric LT. A recent study showed that PV stent placement during the transplant or in the immediate postoperative setting through the inferior mesenteric vein offered both a high feasibility and satisfactory results in pediatric recipients [29].

In the HPB group, there were two procedure-related hemorrhages. One patient received PV stent 43 days after a pancreatoduodenectomy. During the PV stent placement, the patient suffered bleeding from a minor hepatic arterial branch and underwent embolization 8 days later. The patient eventually died in the intensive care unit 48 days after PV stent placement and 91 days after pancreatoduodenectomy due to pneumonia and respiratory failure due to complications after PV stent placement. The other hemorrhage was minor bleeding from the liver capsule that was successfully treated with percutaneous hemostatic applications. Another two patients experienced early stent thrombosis and permanent stent failure with a result comparable to the situation before stent placement. The possible reason for early stent occlusion in the first patient may be postoperative pancreatic fistula causing nearby inflammation and thus promoting thrombosis. The second patient had a recurrence of distal cholangiocarcinoma around the PV with resulting subtotal PV occlusion eight months after bile duct resection. Two days after PV stent placement, early stent thrombosis was confirmed with possible cause being thrombophilia in a palliative patient with advanced metastatic cancer surrounding the PV.

A summary of selected studies reporting PV stent placement for the treatment of PV stenosis after HPB surgery and LT are presented in Table 3. The studies show a technical success rate of 78–100%, a clinical success rate of 72–100%, and a long-term patency of 57–100%. The procedure-related complication rate varied from 0–33.3%. In the current study, PV stent placement was performed on three HPB patients with asymptomatic PV stenosis. Of note, in other published studies, PV stent placement after HPB surgery was only performed in patients with symptoms of PV stenosis such as ascites, gastrointestinal bleeding, or liver dysfunction (Table 3). Thus, it may be questioned if patients with asymptomatic stenosis should undergo PV stenting after HPB surgery. Kang et al. showed that about 20% (162 of 826) of patients undergoing pancreatoduodenectomy developed PV stenosis/occlusion, with a significantly higher rate in patients who underwent PV resection [13]. Moreover, 13% (21 of 162) of the patients with PV stenosis/occlusion developed gastric or hepaticojejunostomy varices. Accordingly, the authors recommended careful postoperative surveillance for PV stenosis/occlusion after pancreatoduodenectomy. However, although 21% (5 of 21) of the patients with gastrointestinal bleeding experienced fatal recurrent bleedings, routine PV stenting is not generally recommended in patients with asymptomatic PV occlusion because the incidence of complications related to PV stenting is seemingly higher than the rate of gastrointestinal bleeding caused by portal hypertension. However, an aggressive approach including PV stenting or selective surgical therapy to lower PV hypertension is recommended in patients who develop recurrent gastrointestinal bleeding. For patients with symptomatic PV stenosis/occlusion caused by unresectable malignant tumors, attempts have been made to identify groups that benefit from PV stenting, but results remain inconclusive mainly due to small study samples [38]. Most of these patients have advanced disease and it is difficult to predict whether PV stent placement can provide a survival benefit in this subgroup of patients.

**Table 3:** Summary of selected studies reporting portal vein stent placement for the treatment of portal vein stenosis after hepato-pancreato-biliary surgery and liver transplantation

Author/year	*N*	Surgery/procedure	Indication for PV stent	Technical success rate	Clinical success rate	Complications	Patency
Funaki 2000/Radiology [30]	12	13 balloon angioplasties alone and 12 successful PV stents (living donor LT)	Postoperative PV stenosis	100%	100%	0%	100% (5–61 months follow-up)
Wang 2006/Transplant Proc [31]	9	9 successful PV stents (LT)	Postoperative PV stenosis	100%	100%	0%	100% (6–19 months follow-up)
Ko 2007/Liver Transpl [23]	9	7 successful PV stents (LT)	Postoperative PV stenosis	78%	78%	33.3%	100% (66.6 months follow-up)
Wei 2009/W. J. Gastroenterol [32]	16	16 successful PV stents (LT)	Postoperative PV stenosis	100%	100%	0%	100% (3.3–56.6 months follow-up)
Woodrum 2009/J Vasc Interv Radiol [33]	18	14 successful PV stents (HBP surgery)	Symptomatic postoperative PV stenosis	78%	72%	0%	Stent patency: 0.2–29 months
Kim 2011/Am J Roentgenol [11]	19	18 successful PV stents (HPB surgery)	Symptomatic postoperative PV stenosis	95%	84%	15.8%	89% (23.5 ± 22.5 months follow-up)
Carnevale 2011/Pediatr Transplant [34]	4	11 balloon angioplasties alone and 4 successful PV stents (pediatric LT)	Postoperative PV stenosis	100%	100%	0%	100% (9–92 months follow-up)
Cao 2013/Acta Radiol [35]	14	13 successful PV stents (HPB surgery and LT)	Symptomatic postoperative PV thrombosis	93%	93%	7%	57% (16.3 months follow-up)
Zhou 2014/ANZ J Surg [28]	59	59 successful PV stents (HPB surgery)	Symptomatic postoperative PV stenosis	100%	89%	8.4%	74.6% (13 ± 11 months follow-up)
Hiyoshi 2015/World J Surg [12]	5	4 successful PV stents (pancreatoduodenectomy)	Symptomatic postoperative PV stenosis	80%	80%	0%	Stent patency 21–41 months
Chang 2016/Kaohsiung J Med Sci [19]	6	6 successful PV stents (LT)	Postoperative PV stenosis	100%	67%	0%	100% (9–55 months follow-up)
Jeon 2016/World J Gastroenterol [24]	22	21 successful PV stents (21 HPB surgeries and 1 LT)	Symptomatic postoperative PV stenosis	95%	95%	0%	97.5% (1.4–25.4 months follow-up)
Shim 2017/Acta Radiol [36]	22	19 successful PV stents (HPB surgery)	Symptomatic postoperative PV stenosis	86%	82%	0%	66.7% (0.2–38.6 months follow-up)
Kato 2017/BMC Surg [37]	29	29 successful PV stents, 22 percutaneous stents and 7 stents via laparotomy (27 HPB surgeries and 2 LT)	Postoperative PV stenosis	100%	81%	0%	76% (17.3 ± 21.4 months follow-up)

Vascular interventional procedures may be contraindicated in the very early postoperative period (< 3 weeks) due to the risk of suture dehiscence and anastomotic bleeding. This did, however, not pose a problem in our study and the shortest time from primary operation to PV stent placement was 21 days. Concerns about the long-term stent patency have been reported [28, 39, 40]. The role of anticoagulation regarding PV stent placement is not well established. Our strategy was prophylactic low molecular weight heparin based on their body weight (150 IU/kg/day) for a minimum of three months. Aspirin was not routinely used; however, patients using aspirin before surgery continued their lifelong regimen throughout the hospital stay. In several studies [8, 9, 22, 40–43], no routine administration of anticoagulation was given as the risk of gastrointestinal bleeding was weighed against portal stent thrombosis. In our experience, the indication for anticoagulation after PV stent placement is very strong in patients with an intravascular thrombogenic foreign body in the PV sometimes combined with a malignant disease. The risk of gastrointestinal bleeding is also significantly reduced after a normal portal flow is reestablished. Our routine administration of anticoagulation may be a possible cause of acceptable long-term stent patency.

The limitations of this study lay in the retrospective design. The study sample was small and the study population was highly heterogeneous with respect to procedures being performed and causes of PV stenosis/occlusion. Nevertheless, we experienced an acceptable short- and long-term outcome. However, it is difficult to draw manifest conclusions and the findings of the study must be interpreted with caution. The literature review revealed that only two of 14 published papers have included more patients (29 and 59, respectively) than the 23 patients in the current study. Thus, it will be difficult to gather a larger collective of patients with this condition within a reasonable time span, even for high-volume referral centers. Management of anticoagulation after stenting and differences between collectives after liver transplant or HPB surgery are topics that should be further investigated. Moreover, a future meta-analysis of published studies on this topic could be useful. In conclusion, our results support the use of percutaneous transhepatic PV stent as a safe and effective treatment for postoperative PV stenosis or occlusion in patients undergoing LT regardless of symptoms. Due to increased risk of complications, the indication for percutaneous PV stent placement after HPB surgery should be limited to patients with clinical symptoms after an individual assessment.
